# Circular RNA circ-*ZKSCAN1* inhibits bladder cancer progression through miR-1178-3p/p21 axis and acts as a prognostic factor of recurrence

**DOI:** 10.1186/s12943-019-1060-9

**Published:** 2019-09-03

**Authors:** Junming Bi, Hongwei Liu, Wei Dong, Weibin Xie, Qingqing He, Zijian Cai, Jian Huang, Tianxin Lin

**Affiliations:** 1 Department of Urology, Sun Yat-Sen Memorial Hospital, Sun Yat-Sen University, 107.W. Yanjiang Road, Guangzhou, Guangdong 510120 People’s Republic of China; 2 Guangdong Provincial Key Laboratory of Malignant Tumor Epigenetics and Gene Regulation, Sun Yat-Sen Memorial Hospital, Sun Yat-Sen University, Guangzhou, People’s Republic of China; 3 Department of Urology, Affiliated Hospital of Guangdong Medical University, Zhanjiang, People’s Republic of China; 4 Department of Urology, Union Hospital, Tongji Medical College, Huazhong University of Science and Technology, Wuhan, People’s Republic of China

**Keywords:** Circ-*ZKSCAN1*, Bladder cancer, miR-1178-3p, p21

## Abstract

**Background:**

Circular RNAs (circRNAs) represent a subclass of regulatory RNAs that have been shown to have significant regulatory roles in cancer progression. However, the biological functions of circRNAs in bladder cancer (BCa) are largely unknown.

**Methods:**

Cell invasion models were established, and invasion-related circRNAs were detected by qPCR. Using above method, circ-*ZKSCAN1* was picked out for further study. Circ-*ZKSCAN1* expression and survival analyses were performed through qPCR. The survival curves were generated by the Kaplan-Meier method, and the log-rank test was used to assess the significance. Cell proliferation, migration and invasion were examined to investigate the function of circ-*ZKSCAN1*. Tumorigenesis in nude mice was assessed to determine the effect of circ-*ZKSCAN1* in bladder cancer. Biotin-coupled probe pull-down assays, FISH and luciferase reporter assays were conducted to confirm the relationship between circ-*ZKSCAN1* and microRNA. RNA-seq revealed different molecular changes in downstream genes.

**Results:**

Here, we found that circ-*ZKSCAN1* was downregulated in BCa tissues and cell lines. Circ-*ZKSCAN1* levels were associated with survival, tumor grade, pathological T stage and tumor recurrence. Overexpressed circ-*ZKSCAN1* inhibits cell proliferation, migration, invasion and metastasis in vitro and in vivo. Mechanistically, we demonstrated that circ-*ZKSCAN1* upregulated p21 expression by sponging miR-1178-3p, which suppressed the aggressive biological behaviors in bladder cancer.

**Conclusions:**

These results reveal that Circ-*ZKSCAN1* acts as a tumor suppressor via a novel circ-*ZKSCAN1*/miR-1178-3p/p21 axis, which have the important role in the proliferation, migration and invasion ablitities of BCa cells and provide a novel perspective on circRNAs in BCa progression.

**Electronic supplementary material:**

The online version of this article (10.1186/s12943-019-1060-9) contains supplementary material, which is available to authorized users.

## Background

Bladder cancer (BCa) is one of the most common urinary malignancies, with approximately 429,800 new cases and 165,100 deaths annually worldwide [1]. BCa is categorized into 2 groups: non-muscle-invasive bladder cancer (NMIBC) and muscle-invasive bladder cancer (MIBC) [2]. NMIBC is the most common BCa and is often treatable [2]. Despite multiple therapeutic approaches have improved, the prognosis for MIBC with extensive invasion and metastasis remains poor [3]. To metastasize, BCa cells must undergo a series of selective steps, including transformation, migration, invasion, vascularization, transportation within the circulatory system, and establishment in different organs [4]. Among these highly selective events, the proliferation, migration and invasion of BCa cells into muscle are crucial early steps [4]. However, the mechanisms of proliferation, migration and invasion have not been fully elucidated. Thus, to improve the treatment of bladder cancer, it is vital to have a better understanding of changes in genes during proliferation, migration and invasion.

Circular RNAs (circRNAs) make up a novel class of regulatory RNAs that are formed by a covalently closed loop [5, 6]. With massively parallel sequencing technology, researchers were able to explore the functions of circRNAs [7]. Previous studies revealed that the expression of circRNAs is cell type- and tissue-specific and can be mostly independent of the host gene [8]. CircRNAs are involved in the progression of many cancers, such as gastric cancer, glioma, and hepatocellular cancer, by being sponges of microRNAs and keeping target genes away from miRNAs [9–11]. Nevertheless, only a few circRNAs are predicted to be able to carry out such “sponging” activities [12]. Moreover, previous studies showed that circRNAs was more stable than mRNAs [9–11]. Besides, some circRNAs could have the potential to be biomarkers or prognostic factors in tumors [13]. However, few circRNAs in BCa have been reported [14, 15]. The functions of circRNAs in BCa remain largely unknown and need to be elucidated.

MicroRNAs (miRNA), a class of small and non-coding RNA, directly regulate a variety of biological processes of mRNAs in multiple cancer types [13]. Increasing evidences demonstrated that miRNAs exert influences on inhibiting or promoting invasion, migration and apoptosis in bladder cancer [16, 17]. For instance, miR-520f could reverse EMT and inhibit metastasis by targeting ADAM9 and TGFBR2 [18]. Mir-129 could exert growth inhibitory effect and induce cell death through targeting GALNT1 and SOX4 [19]. Recently, studies have reported that circRNAs could act as “miRNA sponges” in many cancers [20]. However, the relation between circRNAs and miRNAs has not been clearly elucidated in bladder cancer.

In the present research, we identified circRNAs from previous circRNA microarray data of human BCa tissues and normal bladder tissues [21]. Then we established highly invasive (UM-UC-3-M and T24-M) and lowly invasive cell sublines (UM-UC-3-NM and T24-NM) using a repetitive transwell assay in vitro [21]. We examined the circRNAs expression profile of each cell subline to identify differentially expressed circRNAs related to BCa invasion. Among these, circ-*ZKSCAN1*, a significantly upregulated circRNA in lowly invasive cells, was shown to be closely correlated with BCa tumorigenesis and metastasis in patients. Furthermore, we found that enforced circ-*ZKSCAN1* could suppress BCa progression in vivo and in vitro through a novel circ-*ZKSCAN1*/miR-1178-3p/p21 axis.

## Methods

### Tumor specimens

BCa tissues and the matched normal tissues were obtained from the bladder cancer tissue bank of our institute. All patients were histopathological diagnosed with BCa and undergone radical cystectomy between 2013 and 2017, without neoadjuvant chemotherapy or radiotherapy. Sixty-eight pairs of samples were frozen in liquid nitrogen and extracted by trizol. The grading of BCa was determined according to the histologic tumor grading system of the World Health Organization [22], and BCa staging was examined according to the TNM Classification of Malignant tumors established by the Union for International Cancer Control [23]. The study was approved by the ethical Review Committee of Sun Yat-sen Memorial Hospital. All the patients had signed the informed consent. Additionally, a cohort of 68 BCa patients with histopathological diagnosis were followed up. Patients were followed up till February 2018 and the median follow-up time is 81.3 months.

### Cell culture and transfection

Human Bladder cancer cell lines (T24, UM-UC-3, 5637, EJ, SV-HUC-1) were purchased from ATCC (American Type Culture Collection. Manassas, VA, USA). T24, 5637, EJ and SV-HUVEC Cells were cultured in RPMI-1640 (Gibco, Shanghai, China). While UM-UC-3 was cultured in DMEM (Gibco, Shanghai, China). All the medium was supplemented with 10% fetal bovine serum and 1% penicillin/streptomycin (Gibco, Shanghai, China). Highly metastatic and lowly metastatic BCa cell model were established by the method described by previous study [21]. Eventually, highly invasive cell clones UM-UC-3-M, T24-M, and a lowly invasive clones UM-UC-3-NM, T24-NM were established. The cells were grown in a humidified atmosphere of 5% CO2 at 37 °C. siRNAs and microRNA mimics were purchased from GenePharma (Shanghai, China). Oligos were showed in Additional file 1 Table S1. Transfection was carried out using reagent Lipofectamine RNAiMAX (Thermo Fisher Scientific Inc. Massachusetts, USA) following manufacturer’s protocol.

### CircRNA plasmid construction and stable transfection

To construct circ-ZKSCAN1-overexpressing plasmids, circ-ZKSCAN1 cDNA was synthesized and cloned into the plenti-ciR-GFP-T2A vector (IGE Biotech Co, China). Plasmids were transfected into 293 Tcells to package lentivirus using X-treme (Sigma, USA) according to the manufacturer’s instructions. T24 and UM-UC-3 cells were infected with the packaged lentivirus and selected with 2 μg/ml puromycin for 3 days.

### RNA isolation and quantitative real-time PCR

Total RNA was isolated using the RNAiso Plus (TaKaRa, Japan) according to the manufacturer’s instructions. cDNA was synthesized using the PrimeScript RT Reagent Kit (Takara, China) and microRNA First-Strand cDNA Synthesis Kit (Sangon Biotech, China). The expression of circRNA and mRNA in cancer samples and cell lines was performed on an ABIPRISMVR 7300 Sequence Detection System (Applied Biosystems). Each reaction was performed in triplicate. Additional file 1 Table S1 lists the specific primers.

### Western blotting

Western blotting was performed as previously described [24]. Primary antibodies specific to p21 (1:1000; CST, Danvers, Massachusetts, USA) and GAPDH (1:5000; CST, Danvers, Massachusetts, USA) were used. The blots were then incubated with goat anti-rabbit or anti-mouse secondary antibody (CST) and visualized by commercial ECL kit (Pierce, Rockford, IL).

### MTS assay

BCa cells (1000 cells per well) were seeded into 96-well plates and 20 μl MTS (Promega, Beijing, China) was added to each well for 3 h incubation. And then we measured the absorbance of each well at 492 nm every 24 h for 6 times.

### Cell migration and invasion assay

For cell migration assays, cell suspensions (5 × 10^4^ cells) were seeded to the upper chamber and incubate for 20 h with 1% FBS. For invasion assays, the bottom of the upper chamber was coated with Matrigel (BD Bioscience) and incubate for 24 h.

After the incubation, cells were fixed and stained with crystal violet. Through counting the cells under the microscope, migration and invasion rates were quantified.

### Animal experiments

All animal experiments were approved by the the ethical Review Committee of Sun Yat-sen Memorial Hospital. 4 weeks old Male BALB/c nude mice were purchased from the Experimental Animal Center, Sun Yat-sen University (Guangzhou, China). Mice were randomly divided into two groups: vector and circ-*ZKSCAN1*. Cells (5 × 10^6^/mice) were injected into the foot-pad of right hind or subcutaneously injected into upper back of nude mice. On day 28, the mice were euthanized and primary tumors and popliteal lymph nodes were enucleated and paraffin embedded.

### Immunohistochemistry analysis

All paraffin-embedded tissue sections were examined by immunohistochemistry (IHC) using the experimental procedure as previously described with antibody specific for p21 (1:200; CST, Danvers, Massachusetts, USA). At least two pathologist had assessed the immunoreactivity in each section.

### CeRNA analysis and target prediction

Using the bio-informatic database miRanda and TargetScan, we predicted the miRNA-binding sites of circ-*ZKSCAN1*. The interaction between mRNA and miRNA was predicted by miRanda and TargetScan. Filtering restrictions were as follows: Context Sore ≥90.

### Pull-down assay

The pull-down assay was performed using the procedure as previously described [14, 15]. In brief, T24 cells that stably expressed circ-*ZKSCAN1* were harvested, lysed, and sonicated. To generate the probe coated beads, circ-*ZKSCAN1* probe and NC probe was incubated with magnetic beads. After 2 h incubation, cell lysates were incubated with the probes overnight. After the incubation, the bound RNAs were washed and purified for the analysis. Circ-*ZKSCAN1* biotinylated-probe was designed and synyhesized by GenePharma (Shanghai, China).

### Fluorescence in situ hybridization (FISH)

The Fluorescence in situ hybridization (FISH) assay was performed using the procedure as previously described [25]. In brief, T24 cells were seeded and fixed in confocal dish. After prehybridization and hybridization, cells were incubate with cy3-labelled circ-*ZKSCAN1* probe (GenePharma, China) at 37 °C overnight. For double FISH assay, circ-*ZKSCAN1* overexpressed T24 cells were transfected with miR-1178-3p mimics. After the transfection, cells were hybridized with circ-*ZKSCAN1* probe (cy3-labelled) and miR-1178-3p probe (cy5-labelled). The images were acquired on ZEISS LSM800 Confocal Microscope (Carl Zeiss AG, Germany). Sequences of probes were showed in Additional file 1 Table S1.

### Luciferase assay

Dual-luciferase reporter assay was used to evaluate the direct binding between circ-*ZKSCAN1* and miRNAs. psiCHECK2 (GenePharma, China) vector contains firefly luciferase gene (hLuc+) and renilla luciferase gene (hRluc). The sequence of circ-*ZKSCAN1* was cloned into the vector. NC vector or circ-*ZKSCAN1* vector was co-transfected with each miRNAs mimics. The relative values of hLuc+ and hRluc were detected by Centro LB960 XS3 (Berthold, German). Luciferase reporter assay was used to detect whether p21 is the direct target of miR-1178-3p. The 3’UTR sequence of p21 was cloned into pcDNA3.0 vector. Next, miR-1178-3p mimics or NC was co-transfected with wild-type vector or the mutant vector. The relative value of luciferase was also detected by Centro LB960 XS3 (Berthold, German).

### Statistical analysis

Statistical analysis in our cohorts was carried out with Graphpad prism. The Chi-square test was used to evaluate the association of the expression of circ-*ZKSCAN1* with the patient’s clinicopathological characteristics. Survival curves were assessed with the Kaplan-Meier method and compared by the log rank test. Correlations were analyzed by Pearson’s correlation test. *P* values of < 0.05 were considered significant. The data were presented as means ± Standard Deviation (SD) in the bar charts and were calculated difference by either Student’s t-test or Chi-square test. P value of < 0.05 was considered statistically significant.

## Results

### Identification of invasion-related circRNAs in BCa cells

**Fig. 1:**
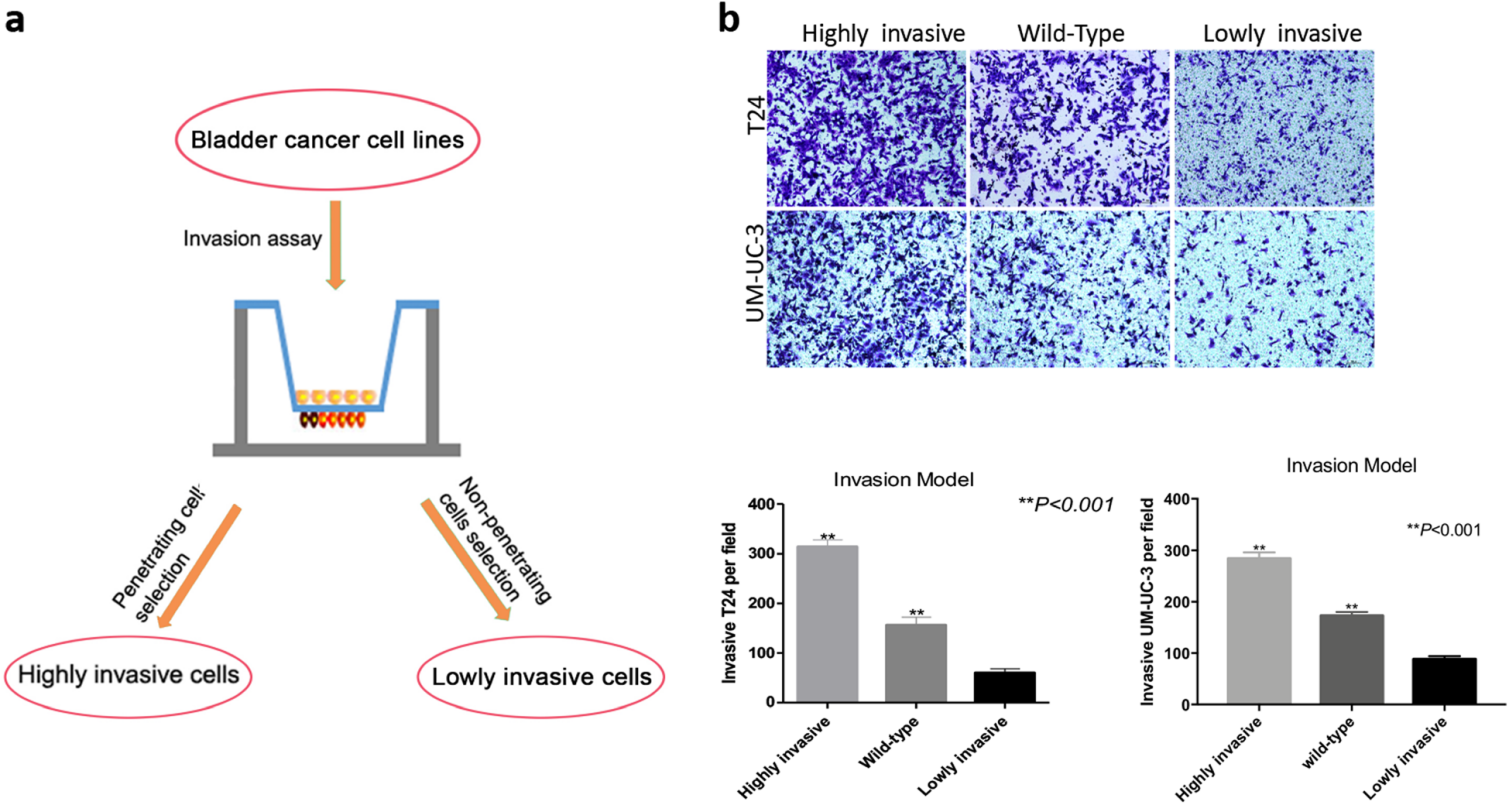
Identification of invasion-related circRNAs in BCa cells. **a** The scheme of the establishment of invasive and noninvasive cell sublines from T24 and UM-UC-3 cell line. **b** Cell invasive abilities of wild-type, highly and lowly invasive bladder cancer cell sublines were evaluated by transwell invasion assays

First, we analyzed and selected 35 differentially expressed circRNAs from circRNAs microarray data of previous study (fold change ≥2.0 or < 0.5 and *P* < 0.05) [25]. Using qPCR, 21 circRNAs were detected in BCa cell lines. (Additional file 2 Table S2). To determine the mechanism of BCa migration and invasion, we established highly invasive BCa cell lines and lowly invasive BCa cell lines using the repeated invasion transwell method (Fig. 1a) [21]. Using this method, we established two lowly invasive sub-clones T24-NM, UM-UC-3-NM, and two highly invasive sub-clones T24-M, UM-UC-3-M (Fig. 1b). Twwenty-one circRNAs were detected in sub-clone cell-lines by qPCR. Fourteen circRNAs were differentially expressed in sub-clone cell-lines by qPCR and found to have effects on invasive abilities of BCa cell lines by transwell migration assays (data not shown).

### Characterization of circ-*ZKSCAN1* in BCa cells

**Fig. 2:**
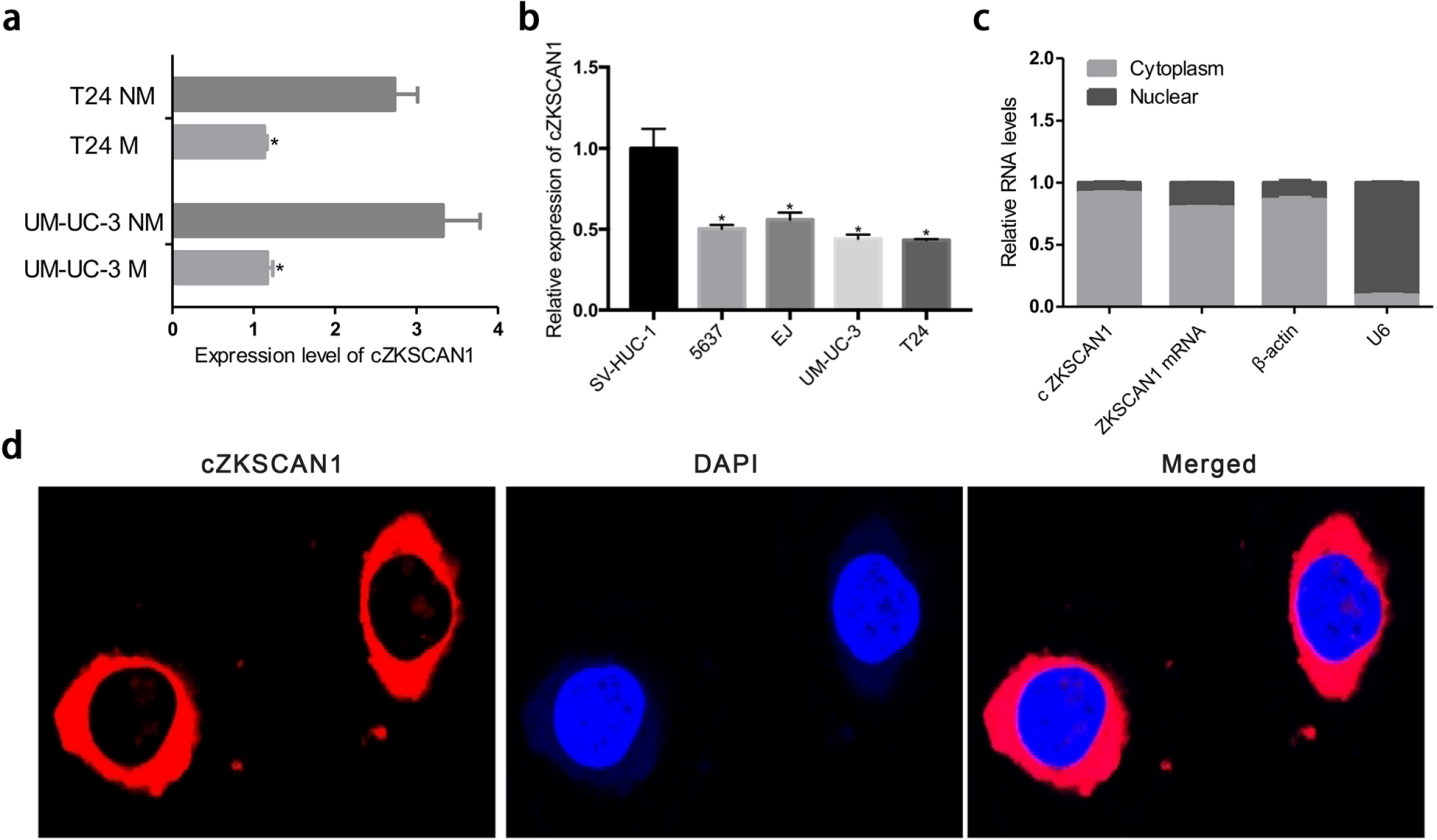
Characterization of Circ-*ZKSCAN1* in BCa cells. **a** The expression of circ-*ZKSCAN1* in wild-type, highly and lowly invasive baldder cancer cell sublines was detected by qPCR. **b** qPCR was used to examine the expression of circ-*ZKSCAN1* in SV-HUC-1, 5637, EJ, UM-UC-3 and T24 cells. **c** Nuclear mass separation assay showed that circ-*ZKSCAN1* was predominantly localized in cytoplasm. β-actin was mostly localized in cytoplasm and U6 was mainly localized in nucleus, which used as a negative control. **d** Fluorescence in situ hybridization (FISH) also showed that circ-*ZKSCAN1* was predominantly localized in cytoplasm. The abbreviations of circ-*ZKSCAN1* is c*ZKSCAN1*

According to the result of qPCR, circRNA_0001727 was significantly upregulated in T24-NM and UM-UC-3-NM. Then, we focused on its clinical characteristics, functionalities, and mechanisms for further study. CircRNA_0001727, termed circ-*ZKSCAN1*, was upregulated 2.73-fold in T24-NM and 3.32-fold in UM-UC-3-NM (Fig. 2a). Additionally, circ-*ZKSCAN1* showed lower expression in 4 BCa cell lines (5637, UM-UC-3, T24, EJ) than in the normal urothelial cell line (SV-HUC-1) (Fig. 2b). Furthermore, through nuclear mass separation assay and FISH, we found that circ-*ZKSCAN1* was predominantly located in the cytoplasm (Fig. 2c, d). Taken together, these results indicate that circ-*ZKSCAN1* is downregulate in BCa and mainly located in cytoplasm.

### Circ-*ZKSCAN1* expression was decreased in BCa and associated with clinical characteristics

**Fig. 3:**
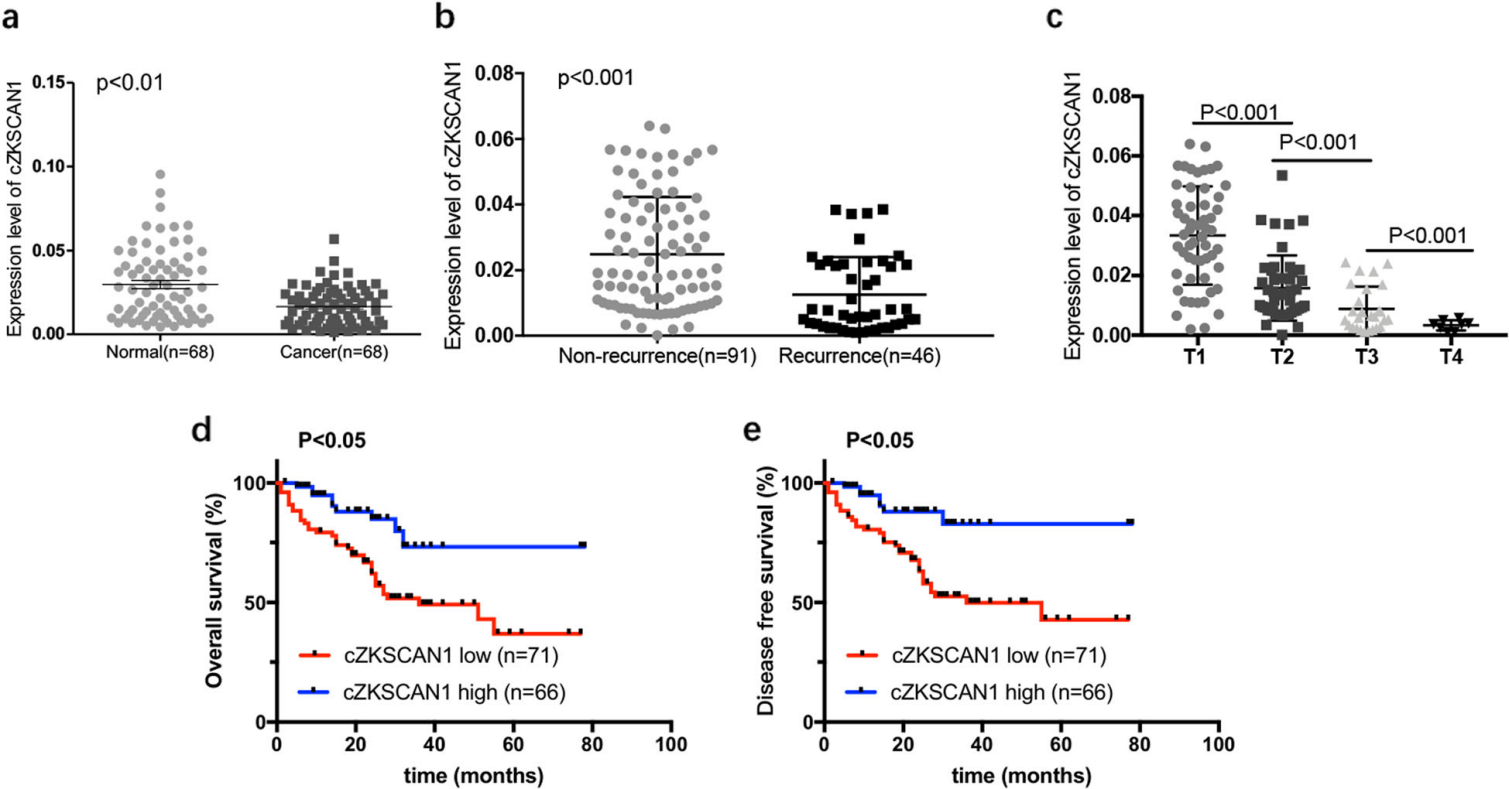
Circ-*ZKSCAN1* expression was decreased in BCa and associated with clinical characteristics. **a** qPCR was used to examine the expression of circ-*ZKSCAN1* in 68 pairs of BCa and normal bladder tissues. β-actin was used us internal control. **b** Expression of circ-*ZKSCAN1* was significantly lower in recurrence patients. **c** Advanced T stage is associated with lower circ-*ZKSCAN1* levels. **d** Kaplan–Meier survival plots showed that lower expression of circ-*ZKSCAN1* leaded to poorer prognosis. **e** Disease-free survival revealed that lower expression of circ-*ZKSCAN1* was associated with shorter time

To investigate whether circ-*ZKSCAN1* was involved in BCa progression, we detected and analyzed circ-*ZKSCAN1* expression in two independent cohorts of BCa tissues and matched adjacent normal tissues. We first performed a qPCR analysis of a 68-cases Cohort 1 of BCa tissues and matched adjacent normal tissues. Compared to the adjacent normal tissues, circ-*ZKSCAN1* expression was significantly downregulated in the BCa tissues (Fig. 3a). While the data from TCGA and Cohort 1 showed that there was no significant change in *ZKSCAN1* mRNA between BCa tissues and matched adjacent normal tissues (Additional file 3 Figure S1a and b).

To further investigate whether circ-*ZKSCAN1* expression was correlated with the clinical features of BCa and patient prognosis, we examined a 137-cases Cohort 2 with complete survival data and clinical characteristic by qPCR. Remarkably, clinicopathological correlation analysis revealed a negative correlation between circ-*ZKSCAN1* levels and recurrence (Fig. 3b). Moreover, statistical analysis revealed that patients with high grade, advanced pathological T stage (Fig. 3c) and positive lymphatic metastasis had lower expression of circ-*ZKSCAN1* (Table 1). However, there was no correlation between circ-*ZKSCAN1* expression and tumor size. Furhermore, Kaplan-Meier survival analysis showed that patients with low circ-*ZKSCAN1* expression were significantly associated with poorer overall survival and disease free survival (Fig. 3d and e). These findings clearly demonstrate that circ-*ZKSCAN1* is downregulated in BCa and lower circ-*ZKSCAN1* expression may lead to a poor prognosis for BCa.

**Table 1:** Correlations between circ-*ZKSCAN1* expression levels and clinicopathological characteristics in BCa

Characteristics	Case	Circ-ZKSCAN1 expression		*P*
		Low (71)	High (66)	value
Age (years)				0.1248
< 65	69	31	38	
≥ 65	68	40	28	
Gender				0.1077
Male	115	56	59	
Female	22	15	7	
Pathology stage				0.0001
pTa-pT1	55	11	44	
pT2-pT4	82	60	22	
Histological grade				0.0001
Low	19	1	18	
High	118	70	48	
Tumor size (cm)				0.8037
< 3	119	61	58	
≥ 3	18	10	8	
Lymph nodes status				0.0038
Negative	106	46	60	
Positive	31	25	6	

Chi-square test. *p < 0.05

### Enforced expression of Circ-*ZKSCAN1* inhibited proliferation, migration, and invasion of BCa cells in vitro

**Fig. 4:**
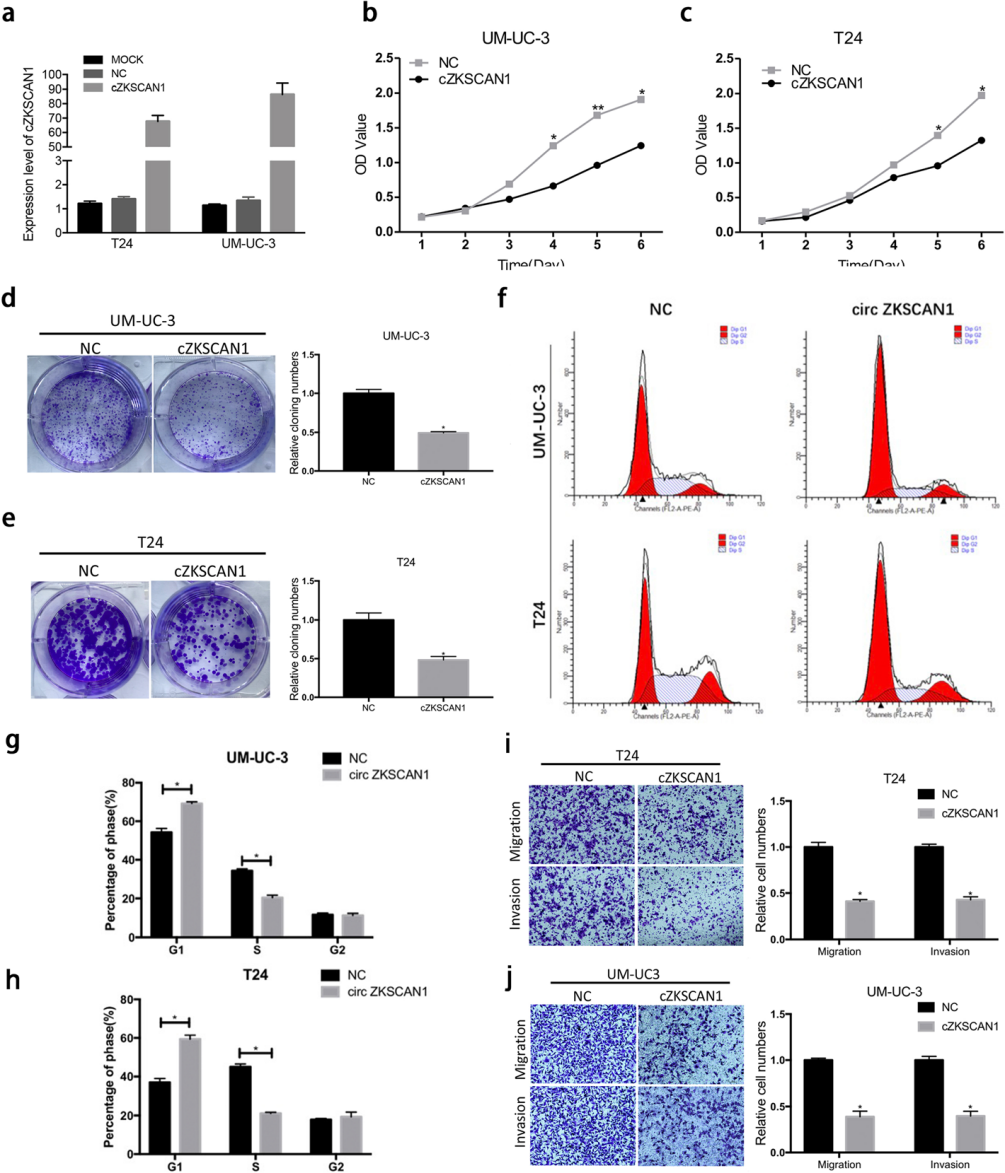
Enforced expression of Circ-*ZKSCAN1* inhibited proliferation, migration, and invasion of BCa cells in vitro. **a** After the overexpression of circ-*ZKSCAN1*, qPCR was used to detect expression of circ-*ZKSCAN1* in T24 and UM-UC-3. **b-c** MTS assays showed that circ-*ZKSCAN1* could inhibite the viablities of T24 and UM-UC-3. **d-e** Clony formation assays demonstrated that circ-*ZKSCAN1* could inhibit the formation of clonies in T24 and UM-UC-3. **f** Representative images of the cell cycle analysis by using flow cytometry. **g-h** Circ-*ZKSCAN1* arrested the UM-UC-3 and T24 cell cycle at the G1/S phase. **i-j** Cell migration and invasion assays were performed and measured in T24 and UM-UC-3 transfected with circ-*ZKSCAN1* or vector

Loss-of-function and gain-of-function assays were performed to evaluate whether circ-*ZKSCAN1* could affect the biological behavior of BCa cells. Since T24 and UM-UC-3 cells expressed the lowest amount of circ-*ZKSCAN1* and EJ, 5637 expressed the highest level of circ-*ZKSCAN1*, they were chosen for further study. We first overexpressed circ-*ZKSCAN1* in UM-UC-3 and T24, and knockdowned circ-*ZKSCAN1* in EJ and 5637. qPCR analysis showed overexpression or knockdown efficiency of circ-*ZKSCAN1* and confirmed that the transfection had no influence on the parental *ZKSCAN1* gene (Fig. 4a, Additional file 4 Figure S2a). Compared to the negative control (NC) group, MTS assays revealed that the viability of UM-UC-3 and T24 decreased in the overexpressed group (Fig. 4b, c). Moreover, colony formation assays showed that proliferative abilities of UM-UC-3 and T24 were also inhibited in the overexpressed group (Fig. 4d, e). Furthermore, Flow cytometry analysis was performed to detect whether circ-*ZKSCAN1* could affect BCa cells phenotype by altering the cell cycle. As shown in Fig. 4f, g and h, after overexpression of circ-*ZKSCAN1*, more cells were distributed in G1 phase, which suggested that circ-*ZKSCAN1* might induce G1/S cell cycle arrest. Additionally, transwell migration and invasion demonstrated that the number of cells was fewer in the overexpressed group than in the NC group (Fig. 4i, j).

In contrast, knocking down circ-*ZKSCAN1* promoted the proliferation, invasion and migration of BCa cells (Additional file 4 Figure S2 b-d). Collectively, these data suggested that enforced circ-*ZKSCAN1* could inhibit the progression of BCa cells in vitro.

### Circ-*ZKSCAN1* may function as a sponge of miR-1178-3p

**Fig. 5:**
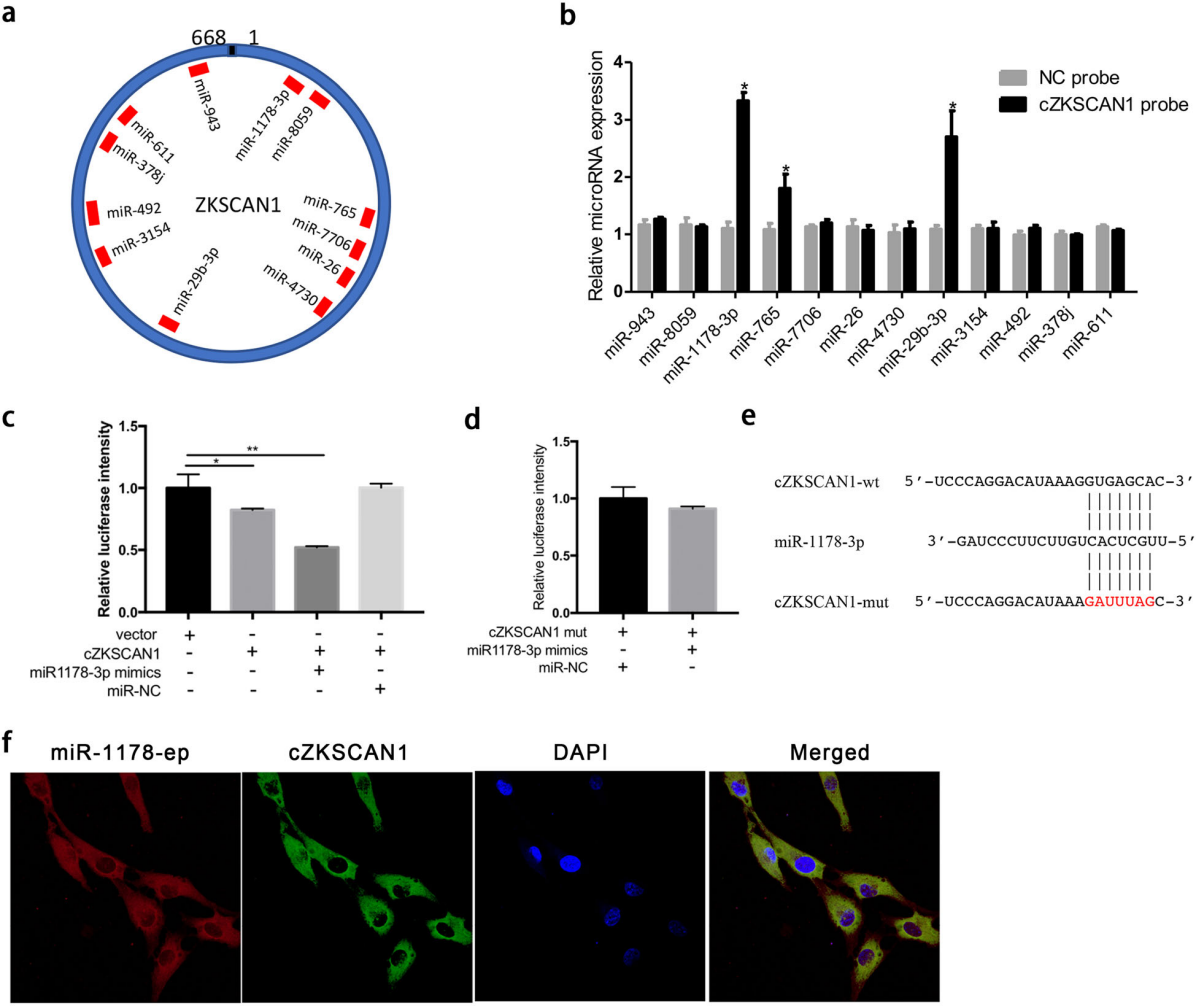
Circ-*ZKSCAN1* may function as a sponge of miR-1178-3p. **a** Schematic model shows the putative binding sites of 12 miRNA candidates correlated with circ-*ZKSCAN1* through miRanda and TargetScan. **b** The relative expressions of 12 miRNAs in UM-UC-3 was detected by qPCR. miR-1178-3p, miR-29b-3p and miR-765 was significantly enriched in the probe of circ-*ZKSCAN1*. **c** Luciferase reporter assay in HEK293T cells co-transfected with miR-1178-3p mimics, empty vector or the vector contained sequence of circ-*ZKSCAN1*. Data were calculated by hRluc to hLuc+. **d** Luciferase reporter assay in HEK293T cells co-transfected with miR-1178-3p mimics and psiCHECK-2-mutant type circ-*ZKSCAN1* (circ-*ZKSCAN1*-mut) plasmids. Data were calculated by hRluc to hLuc+. **e** Schematic of *circ-ZKSCAN1* wild-type (wt) and mutant (mut) luciferase reporter vectors. **f** FISH assay showed that circ-*ZKSCAN1* was co-localized with miR-1178-3p in cytoplasm

Given that circ-*ZKSCAN1* is located in the cytoplasm, we attempted to determine whether circ-*ZKSCAN1* could sponge miRNAs. According to the microRNA response element (MRE) analysis, we used miRanda and TargetScan prediction tool to identify 12 miRNAs that could bind to circ-*ZKSCAN1* (Fig. 5a). Next, we used a biotin-coupled probe pull-down assay to confirm this prediction. The biotinylated circ-*ZKSCAN1* probe and oligo probe were designed and were applied to perform RNA pull-down assay. The pull-down efficiency was verified in UM-UC-3 cells transfected with circ-*ZKSCAN1* or vector. As shown in Fig. 5b, the specific enrichments of miR-1178-3p, miR-29b-3p and miR-765 that were detected in the circ-*ZKSCAN1* pull-down pellet was significantly higher than NC group.

To further confirm this result, we conducted a luciferase assay using mimics of miR-1178-3p, miR-29b-3p and miR-765 (Additional file 5 Figure S3a). We co-transfected each mimics and a luciferase reporter containing sequence of circ-*ZKSCAN1* into HEK293T cells. Compared to the NC, only miR-1178-3p could significantly reduce the luciferase activity (Fig. 5c). Additionally, we found that the co-transfected miR-1178-3p mimics and mutant reporter had no effect on luciferase activity (Fig. 5d, e). We further performed FISH assay to confirm the co-localization of circ-*ZKSCAN1* and miR-1178-3p in the cytoplasm (Fig. 5f). Collectively, these results implied that circ-*ZKSCAN1* could function as a sponge through targeting miR-1178-3p.

### MiR-1178-3p exerts an oncogenic role and partially alleviates effect of circ-*ZKSCAN1* in BCa

**Fig. 6:**
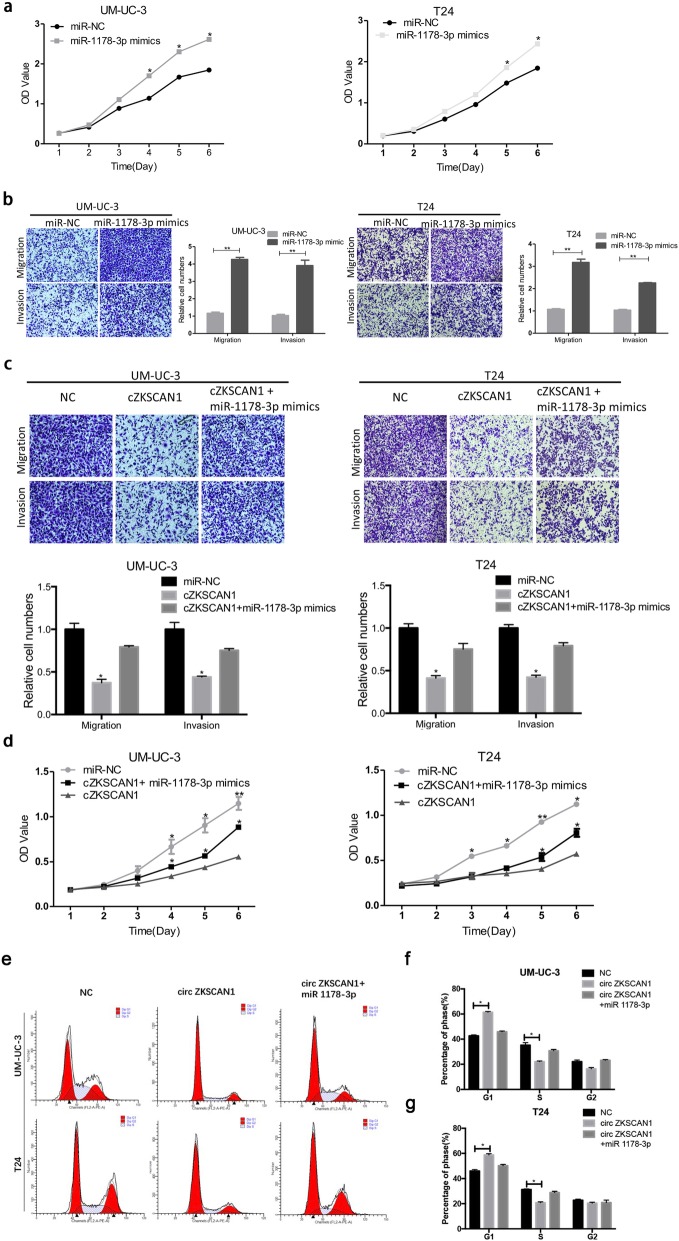
MiR-1178-3p exerts an oncogenic role and partly alleviates effect of *circ-ZKSCAN1* in BCa. **a-b** Transfection of miR-1178-3p mimics enhace the abilities of proliferation, migration and invasion in UM-UC-3 and T24. **c-d** miR-1178-3p could partially reverse the anti-oncogenic effects of circ-ZKSCAN1 on migration, invasion and proliferation in UM-UC-3 and T24. **e** Representative images of the cell cycle analysis by using flow cytometry. **f-g** Circ-ZKSCAN1 arrested the UM-UC-3 and T24 cell cycle at the G1/S phase, while miR-1178-3p could partially turned the G1/S transition

MiR-1178-3p is an oncogene in baldder cancer reported by Liu et al. [26]. In the present study, we found that the expression of miR-1178-3p was upregulated in 56 pairs of BCa tissues compared to the adjacent normal tissues (Additional file 6 Figure S4a). Furthermore, miR-1178-3p was also upregulated in BCa cell lines (Additional file 6 Figure S4b). After the transfection mimics of miR-1178-3p into T24 and UM-UC-3, the abilities of migration, invasion and proliferation were significantly enhanced (Fig. 6a, b). Moreover, rescue experiments were conducted by co-transfecting miR-1178-3p mimics and circ-*ZKSCAN1* in T24 and UM-UC-3. Remarkably, miR-1178-3p mimics could partially alleviate the tumor-suppressing effect of circ-*ZKSCAN1* (Fig. 6c, d, e, f, g).

Overall, these results suggest that circ-*ZKSCAN1* may inhibit BCa cells progression partially through impairing the oncogenic role of miR-1178-3p.

### Circ-*ZKSCAN1* downregulates p21 expression by partly sponging miR-1178-3p

**Fig. 7:**
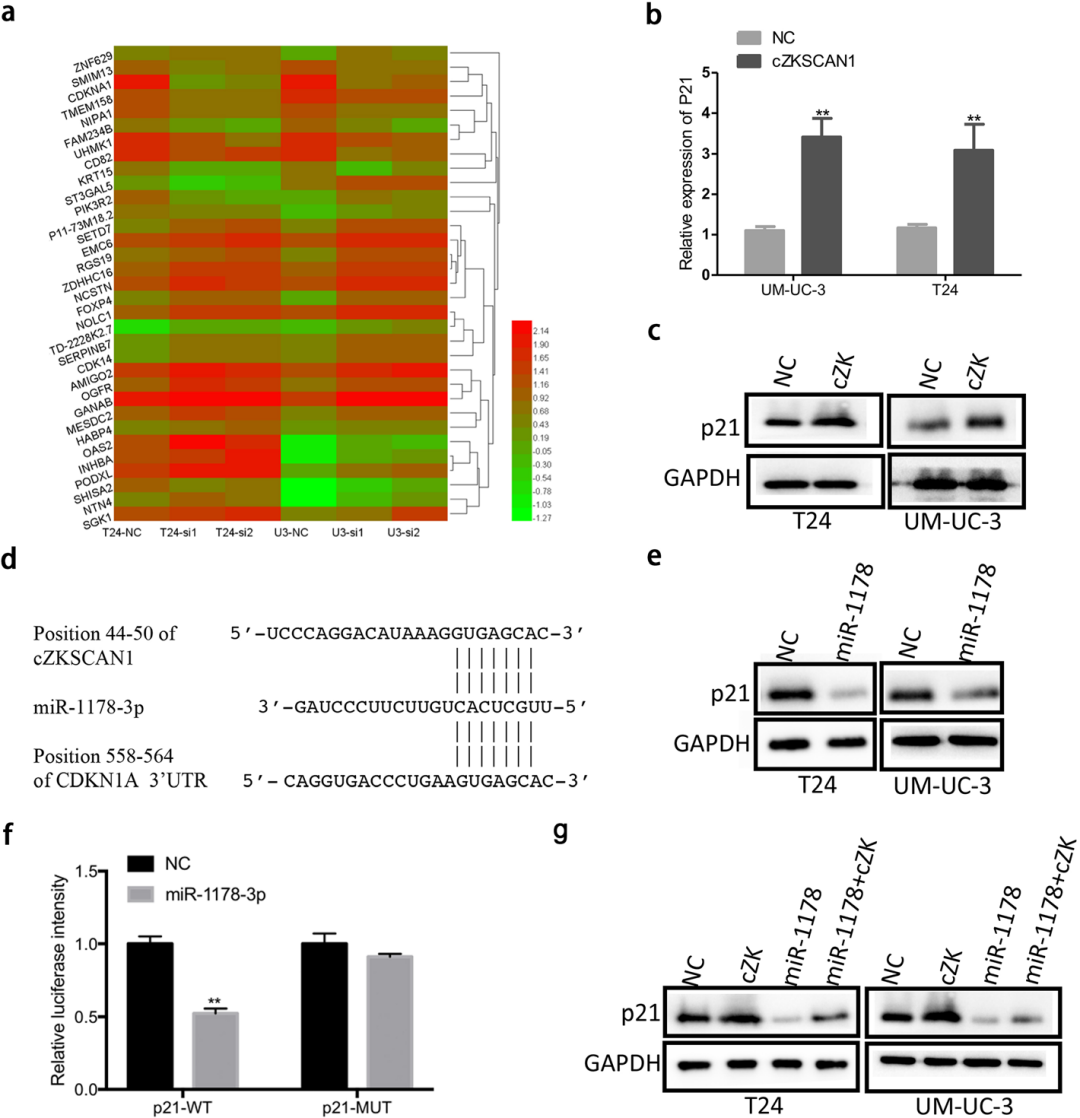
Circ-*ZKSCAN1* downregulates p21(CDKN1A) expression by partially sponging miR-1178-3p. **a** Differentially expressed genes between the Negative Control (NC) and siRNAs groups were showed in the heatmap. Red indicates upregulated expression; green indicates downregulated expression. **b-c** qPCR and western blotting showed that enforced circ-*ZKSCAN1*(cZK) increased the expression of p21(CDKN1A) at both the mRNA and protein levels in UM-UC-3 and T24. **d** The schematics revealed that circ-*ZKSCAN1*(cZK) and p21 shared the same binding sites of miR-1178-3p. **e** Western blotting showed that p21 was downregulated in UM-UC-3 and T24 after transfection of miR-1178-3p mimics. **f** The dual-luciferase reporter assays revealed that luciferase activities was decreased in HEK293T cells after co-transfection with miR-1178-3p mimics and p21-wild-type (p21-wt) vector or p21-mutant (p21-mut) vector. **g** p21 protein expression levels were detected by western blotting after transfected miR-1178-3p mimics or co-transfected miR-1178-3p and circ-*ZKSCAN1*(cZK). The abbreviations of circ-*ZKSCAN1,* miR-1178-3p mimics and CDKN1A are cZK, miR-1178 and p21, respectively

To explore the molecular mechanism of circ-*ZKSCAN1* in BCa, we performed a RNA-seq analysis. Among all genes that are regulated by circ-*ZKSCAN1*, multiple genes play a vital role in the proliferation and invasion of BCa. As shown in Fig. 7a, p21 (CDKN1A) was significantly downregulated in circ-*ZKSCAN1*-silenced cells. To further confirm this result, qPCR and western blotting were performed. The expression of p21 at both the mRNA and protein levels was upregulated in circ-*ZKSCAN1*-overexpressed cells. In total (Fig. 7b, c), these results indicated that circ-*ZKSCAN1* suppressed the proliferation and invasion of BCa cells through regulation of p21.

According to the MRE analysis, miR-1178-3p might target p21 (Fig. 7d). After the transfection of miR-1178-3p mimics, the expression of p21 was significantly reduced at the protein level (Fig. 7e). Additionally, a dual luciferase reporter assay was performed. The wild-type (WT) 3′-UTR sequence of p21 was cloned into the pcDNA3 vector. Co-transfection of miR-1178-3p mimics and p21 wild-type (wt) plasmids strongly reduced luciferase activity by over 41% (Fig. 7f). However, co-transfection of p21 the mutated vector and miR-1178-3p mimics had no obvious effect on luciferase activity (Fig. 7f). Moreover, miR-1178-3p could partially rescue the effect of circ-*ZKSCAN1* on the expression of p21 (Fig. 7g).

Taken together, these results revealed that circ-*ZKSCAN1* suppressed the progression of BCa partially through the miR-1178-3p /p21 axis.

### Overexpression of Circ-*ZKSCAN1* suppresses proliferation and invasion of BCa cells in vivo

**Fig. 8:**
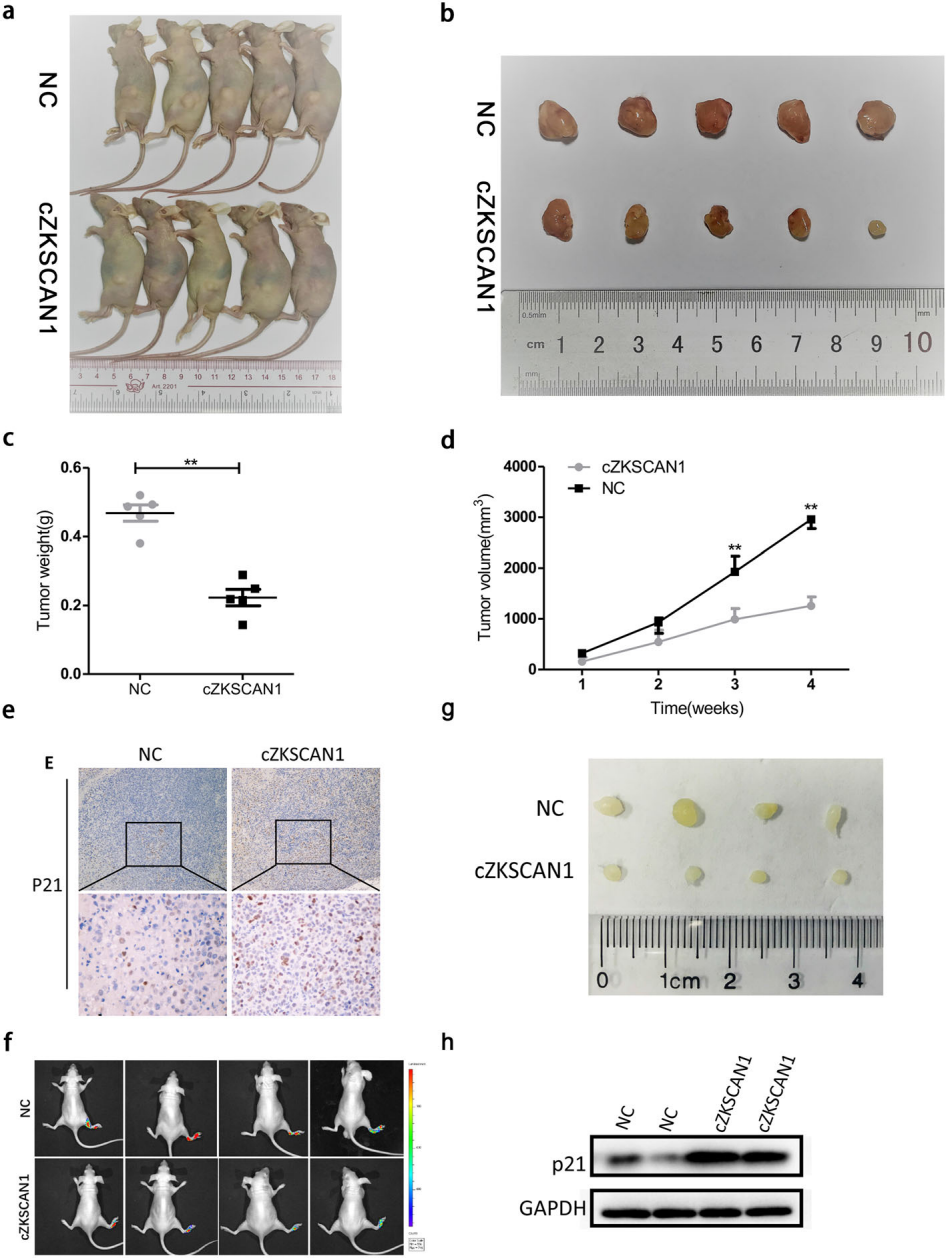
Overexpression of Circ-*ZKSCAN1* Suppresses Proliferation Growth and Invasion of BCa cells in vivo. **a-d** UM-UC-3 stably expressed circ-*ZKSCAN1* or wild-type UM-UC-3 were injected into BALB/C nude mice. (*n* = 5 for each group) Compare with NC group, tumor weight and volume significantly reduced in overexpressed group. **e** IHC revealed that the expression of p21 significantly enhanced in overexpressed group, compare to NC group. **f-g** The luciferase images demonstrated that enforced circ-*ZKSCAN1* could inhibited metastasis in the foot pads model. The popliteal lymph nodes was smaller in overexpressed group than in the NC group. (*n* = 4 for each group) **h** Western blotting revealed that over-expression of circ-*ZKSCAN1* led to increased expression of p21 within xenografted tumors

To explore the effects of circ-*ZKSCAN1* on BCa tumorigenesis in vivo, circ-*ZKSCAN1* was overexpressed in UM-UC-3. Cell from the circ-*ZKSCAN1*-overexpressed and NC groups were subcutaneously injected into BALB/c nude mice, and the tumor volumes were measured weekly (Fig. 8a, b). The sizes and weights of the tumors from the circ-*ZKSCAN1*-overexpressed group were significantly smaller than those of the control group (Fig. 8c, d). These subcutaneous tumors were further subjected to immunohistochemical staining. The expression of p21 was significantly upregulated in the circ-*ZKSCAN1*-overexpressed group compared to the NC group (Fig. 8e). To investigate the role of circ-*ZKSCAN1* in tumor metastasis, circ-*ZKSCAN1*-overexpressed/luc cells and wild-type/luc cells were injected into the foot pads of BALB/c nude mice. The bioluminescence of the popliteal LNs was either undetectable in the circ-*ZKSCAN1*-overexpressed group or dramatically lower than in the NC group (Fig. 8f and g). Western blotting was applied to detect the protein expression of p21 in xenografted tumors of each group. Compare to the NC group,the expression of p21 was obviously increased in tumor tissues of circ-*ZKSCAN1*-overexpressed group (Fig. 8h). Taken together, these data indicated that circ-*ZKSCAN1* inhibited the progression of BCa in vivo.

## Discussion

Currently, a large class of circRNAs have been identified by massively parallel sequencing technology [7]. Previous studies revealed that the expression of circRNAs is cell type- specific and tissue-specific and circRNAs have been proven to be potential biomarkers [8]. CircRNAs are involved in the progression of many cancers by being sponges of microRNAs and keeping target genes away from miRNAs [9–11]. However, the functions of circRNAs in BCa remain largely unkown. In this study, we first analyzed and selected diferentially expressed circRNAs from microarray data [9–11]. Then, we established a BCa cell invasion model and screened invasion related circRNAs. Among the aberrantly expressed circRNAs, circ-*ZKSCAN1* was significantly upregulated in normal tissues and lowly invasive cell subline. Circ-*ZKSCAN1* was first reported by Liang and Wilusz, who showed that circ-*ZKSCAN1* was partially abundant in human brain and liver [27]. Yang found that circ-*ZKSCAN1* was downregulated in hepatocellular carcinoma and acted as a tumor suppressor [28]. To our knowledge, this is the first report on the expression and regulatory function of circ-*ZKSCAN1* in BCa.

Interestingly, the expression of circ-*ZKSCAN1* was dramatically downregulated not only in BCa but also in BCa cell lines. Additionally, we found that lower expression of circ-*ZKSCAN1* was significantly associated with poorer disease free survival, higher recurrence and a positive tumor metastasis status, suggesting its tumor-suppressive effect on BCa. In functional studies, cell proliferation, clone formation, migration and invasion were significantly inhibited in the circ-*ZKSCAN1*-overexpressed group compared with the NC group. Moreover, to explore the effects of circ-*ZKSCAN1* in vivo, circ-*ZKSCAN1*-overexpressed UM-UC-3 and wild-type UM-UC-3 cells were injected into the back and foot pads of nude mice. Strikingly, the growth and metastasis of tumors from circ-*ZKSCAN1*-overexpressed UM-UC-3 were significantly inhibited. Taken together, these findings indicate that circ-*ZKSCAN1* is a tumor suppressor in BCa.

An increasing number of circRNAs that possess the ability to bind to miRNAs has been reported. For example, circ*-ITCH* binds to miR-17 in colorectal cancer and lung cancer [14]. Another circRNA termed circ-*SMARCA5* was observed to be a sponge for miR-17 and miR-181b [11]. In our study, we used the miRanda and TargetScan prediction tool to screen oncogenic miRNAs. Finally, through a biotin-coupled probe pull-down assay and dual luciferase assay, miR-1178-3p was selected and confirmed to be the target miRNA of circ-*ZKSCAN1*. MiR-1178-3p, an oncogene reported by Cao Z. et al., targets tumor suppressors in pancreatic cancer [29]. Further experiments have confirmed that miR-1178-3p promotes the progression of BCa and is upregulated in BCa. To explore the underlying mechanism by which circ-*ZKSCAN1* regulates proliferation and invasion in BCa, we performed RNA-seq to identify changes in gene expression after silencing circ-*ZKSCAN1*. The expression of p21 changed significantly and was confirmed by qPCR and western blot. As a well-known tumor suppressor, p21 can inhibit the growth and metastasis of multiple cancer types [24]. Therefore, we predicted that p21 might be the target gene of circ-*ZKSCAN1* and miR-1178-3p. According to the bioinformatics analysis, dual luciferase reporter assays and rescue experiments, we verified a novel regulatory axis that was formed by circ-*ZKSCAN1*/miR-1178-3p/p21 in BCa.

## Conclusion

In summary, we first analyzed and selected circRNAs from circRNA microarray. Then, we have established BCa cell invasion model and found that circ-*ZKSCAN1* is significantly downregulated in BCa tissues and highly invasive BCa cell lines. This is the first study to explore the profile and mechanism of circ-*ZKSCAN1* in BCa. Circ-*ZKSCAN1* is downregulated in BCa and correlates with tumor metastasis status, recurrence, pathological T stage and histological grade. Additionally, patients with lower circ-*ZKSCAN1* expressions might have poorer prognosis. In functional and mechanistic assays, circ-*ZKSCAN1* suppresses BCa progression in vivo and in vitro through a novel circ-*ZKSCAN1*/miR-1178-3p/p21 signaling regulatory network. Taken together, these data indicated that circ-*ZKSCAN1* might be a potential biomarker and prognostic factor for BCa.

## Additional files

Additional file 1: **Table S1** The sequences of primers, oligonucleotides and probes used in this study. (PDF 47 kb)

Additional file 2: **Table S2** Identification of differentially expressed circRNAs in BCa cells (PDF 10 kb)

Additional file 3: ** Figure S1** Data from TCGA and our cohort showed the expression of *ZKSCAN1* mRNA between BCa tissues and paired adjacent normal tissues. **a** Data from TCGA revealed that there was no significant changes in *ZKSCAN1* mRNA between BCa tissues and matched adjacent normal tissues. **b** qPCR analysis for *ZKSCAN1* mRNA in our cohort 1. (TIF 9877 kb)

Additional file 4: **Figure S2** circ-ZKSCAN1 silencing promotes proliferation, migration and invasion of BCa cells in vitro. **a** qPCR analysis for circ-*ZKSCAN1* and *ZKSCAN1* mRNA in EJ and 5637 cells treated with siRNAs or transfected with negative control. **b-c** The effect of si- circ-*ZKSCAN1* on cell proliferation of EJ and 5637 cells was assessed by colony formation assays. **d-e** The cell migratory and invasive capabilities were examined in EJ and 5637 cells treated with circ-*ZKSCAN1* siRNAs using transwell migration and invasion assays. (TIF 11349 kb)

Additional file 5: **Figure S3** Circ-*ZKSCAN1* may function as a sponge of miR-1178-3p **a** The Renilla luciferase activity of wild type circ-*ZKSCAN1* in the miR-29b-3p/miR-765 mimics or NC group. (TIF 3244 kb)

Additional file 6: **Figure S4** MiR-1178-3p exerts an oncogenic role in BCa **a-b** qPCR revealed that miR-1178-3p was up-regulated in BCa tissues (*n* = 56) and cell lines (UM-UC-3 and T24), compared to the paired normal bladder tissues and SV-HUC-1. (JPG 447 kb)

## Data Availability

The circRNA microarray data of BCa tissues and matched normal tissues analysed during this study are included in this published article and its supplementary information files (PMID: 27050392, PMCID: PMC4823868, 10.1038/ncomms11215). The rest of datasets used and/or analysed during the current study are available from the corresponding author on reasonable request.

## Abbreviations

BCa: Bladder cancer
CircRNAs: Circular RNAs
cZKSCAN1(cCK): circular RNA ZKSCAN1
LN: Lymph node
M: Highly invasive cell line
MRE: microRNA response element
NM: Lowly invasive cell line
p21 (CDKN1A): cyclin-dependent kinase inhibitor 1A
qPCR: quantitative real-time polymerase chain reaction
ZKSCAN1: Zinc finger with KRAB and SCAN domains 1
