# Correction to: Circular RNA circ-ZKSCAN1 inhibits bladder cancer progression through miR-1178-3p/p21 axis and acts as a prognostic factor of recurrence

**DOI:** 10.1186/s12943-020-01265-8

**Published:** 2020-10-12

**Authors:** Junming Bi, Hongwei Liu, Wei Dong, Weibin Xie, Qingqing He, Zijian Cai, Jian Huang, Tianxin Lin

**Affiliations:** 1 Department of Urology, Sun Yat-Sen Memorial Hospital, Sun Yat-Sen University, 107.W. Yanjiang Road, Guangzhou, Guangdong 510120 People’s Republic of China; 2 Guangdong Provincial Key Laboratory of Malignant Tumor Epigenetics and Gene Regulation, Sun Yat-Sen Memorial Hospital, Sun Yat-Sen University, Guangzhou, People’s Republic of China; 3 Department of Urology, Affiliated Hospital of Guangdong Medical University, Zhanjiang, People’s Republic of China; 4 Department of Urology, Union Hospital, Tongji Medical College, Huazhong University of Science and Technology, Wuhan, People’s Republic of China

**Correction to: Mol Cancer 18, 133 (2019)**

**https://doi.org/10.1186/s12943-019-1060-9**

Following the publication of the original article [1], it has been found that Fig. 6c has an error of duplication and Fig. 7c has a misplacement. As shown in the original Fig. 6b, the “UM-UC-3 invasion” images of “miR-1178-3p mimics” was shown identical to the “UM-UC-3 invasion” images of “cZKSCAN1 + miR-1178-3p mimics” in Fig. 6c. The authors found this mistake happened in the process of figure layout. The “T24 invasion” images of “cZKSCAN1” has an error of misplacement in Fig. 6c which cause by that the folder was poorly managed and the process of numerous figure layout. In Fig. 7c, the “P21” images of “T24 cell line” had been misplaced. The mistakes were caused by unintentionally covering the correct image during figure preparation. The mistakes have been corrected in the revised file Fig. 6c and Fig. 7c below. Authors apologize to the editor, reviewers and readers for any inconvenience that caused by these unintentional mistakes.

**Fig. 6:**
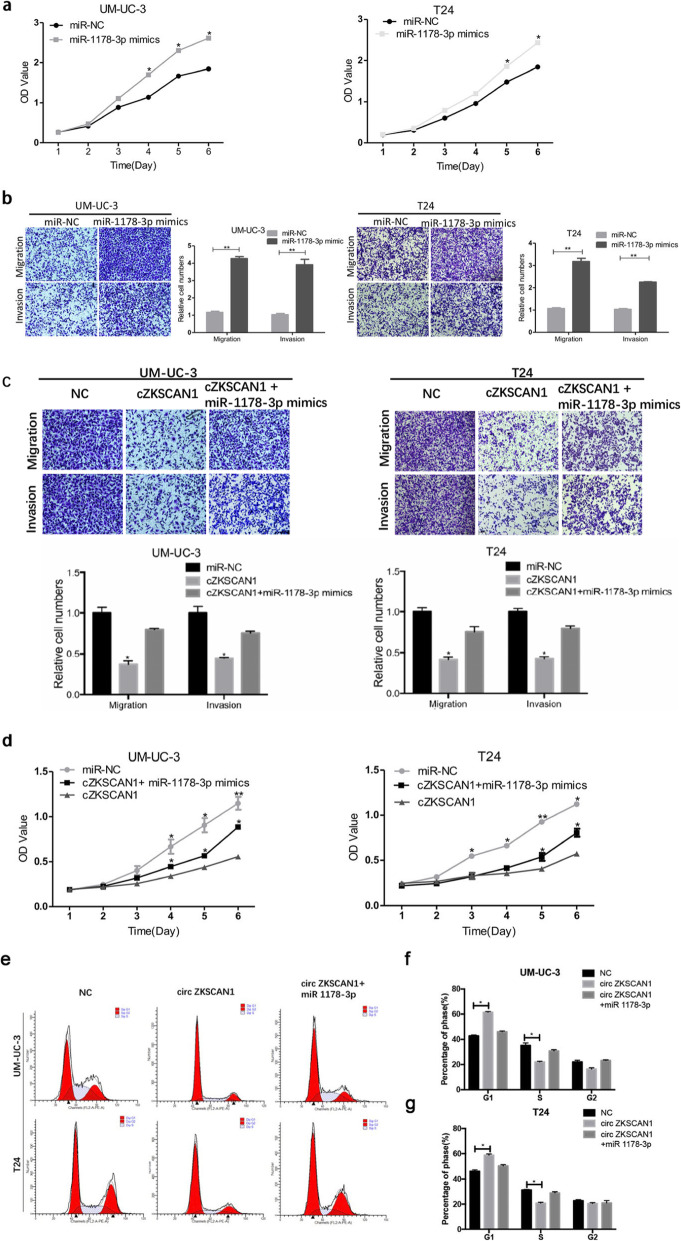
.

**Fig. 7:**
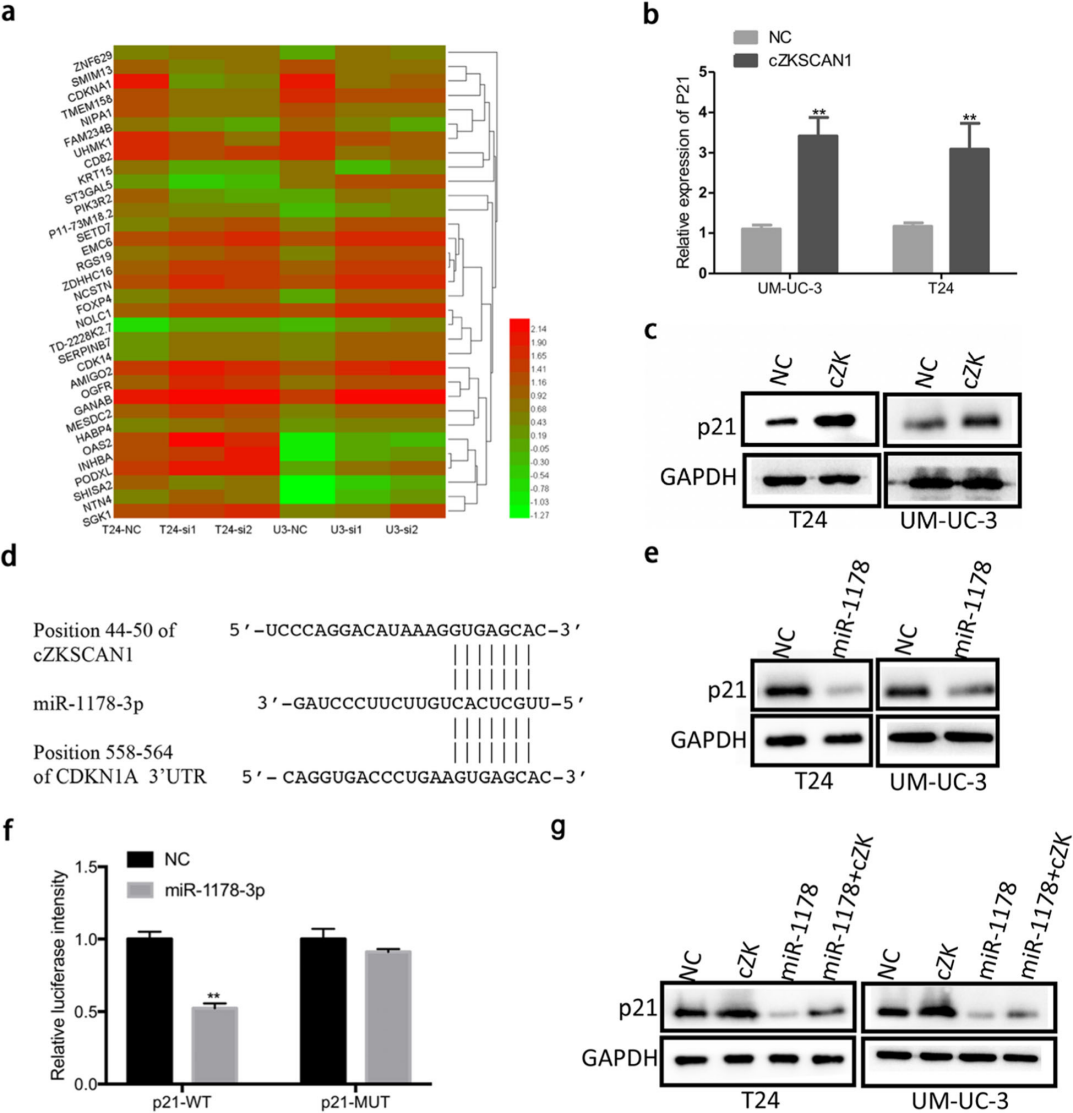
.
