# Supplementary material for: Circular RNA circ-ZKSCAN1 inhibits bladder cancer progression through miR-1178-3p/p21 axis and acts as a prognostic factor of recurrence
Source: Mol Cancer. 2019 Sep 3;18:133. doi: 10.1186/s12943-019-1060-9 (PMC6721182; doi:10.1186/s12943-019-1060-9)
Supplement: Supplementary file 1 — Table S1 The sequences of primers, oligonucleotides and probes used in this study. (PDF 47 kb) [file 12943_2019_1060_MOESM1_ESM.pdf]

Supplementary Table 1.

Gene	Sequence
**Primers for qPCR**	
Circ-ZKSCAN1	5’CCCAGTCCCACTTCAAACAT 3’
	5’TCATTCAGGCTCCAGGAACT 3’
ZKSCAN1	5’ CGCTTCAGGCGCTTCTGTTA 3’
	5’ CCACTATCGGGGCGGTATTC 3’
GAPDH	5’ GGAGCGAGATCCCTCCAAAAT 3’
	5’ GGCTGTTGTCATACTTCTCATGG 3’
miR-1178-3p	5’ TGCTCACTGTTCTTCCCT 3’
p21	5’ TGTCCGTCAGAACCCATGC 3’
	5’AAAGTCGAAGTTCCATCGCTC3’
**RNA oligos**	
Circ-ZKSCAN1 siRNA#1	5’GTCACGAGGAATAGTAAAGAATT 3’
Circ-ZKSCAN1 siRNA#2	5’ACGAGGAATAGTAAAGAAACATT 3’
miR-1178-3p	5’UUGCUCACUGUUCUUCCCCUA
	GAGGGAAGAACAGUGAGCAAUU 3’
**FISH probe sequence**	
Circ-ZKSCAN1	5’TGTTTCTTTATCTATTCCTCGTGACTGTAAGAGGC3’
miR-1178-3p	5’TGTTTCTTTATCTATTCCTCGTGACTGTAAGAGGC3’
U6	5’TTTGCGTGTCATCCTTGCG’
**RNA pulldown biotin probe**	
Circ-ZKSCAN1	5’TGTTTCTTTATCTATTCCTCGTGACTGTAAGAGGC3’
miR-1178-3p	5’UUGCUCACUGUUCUUCCCUAG3’
NC	5’CUGUACUUGUUCCAACUCAAGUGCUAUACUUGGUAGAUCAGA3’
