# Supplementary material for: Circular RNA circ-ZKSCAN1 inhibits bladder cancer progression through miR-1178-3p/p21 axis and acts as a prognostic factor of recurrence
Source: Mol Cancer. 2019 Sep 3;18:133. doi: 10.1186/s12943-019-1060-9 (PMC6721182; doi:10.1186/s12943-019-1060-9)
Supplement: Supplementary file 2 — Table S2 Identification of differentially expressed circRNAs in BCa cells (PDF 10 kb) [file 12943_2019_1060_MOESM2_ESM.pdf]

circRNA	Chromatin	FoldChange	regulation in Bca tissues
circSLC38A1	chr12	0.483679438	down
circZKSCAN1	chr7	0.071214636	down
circPLEKHH2	chr2	0.050453619	down
circVPS13C	chr15	0.232506804	down
circATP8A1	chr4	0.101273285	down
circRHBDD1	chr2	0.196106467	down
circEPB41L2	chr6	0.109356056	down
circZNF292	chr6	0.272838732	down
circTMEM71	chr8	0.109554965	down
circSBNO1	chr12	0.053107738	down
circZFY	chrY	0.252721766	down
circASH1L	chr1	0.395869743	down
circMAN2A1	chr5	0.151760741	down
circPICALM	chr11	0.444882453	down
circADGRL3	chr4	0.133885016	down
circNHLRC2	chr10	0.032039629	down
circZNF483	chr9	0.173002942	down
circRNF13	chr3	14.07356104	up
circGDAP2	chr1	23.14608816	up
circASXL1	chr20	3.942928737	up
circPHC3	chr3	10.55601098	up
