# Supplementary figures and images for: Circular RNA circ-ZKSCAN1 inhibits bladder cancer progression through miR-1178-3p/p21 axis and acts as a prognostic factor of recurrence
Source: Mol Cancer. 2019 Sep 3;18:133. doi: 10.1186/s12943-019-1060-9 (PMC6721182; doi:10.1186/s12943-019-1060-9)

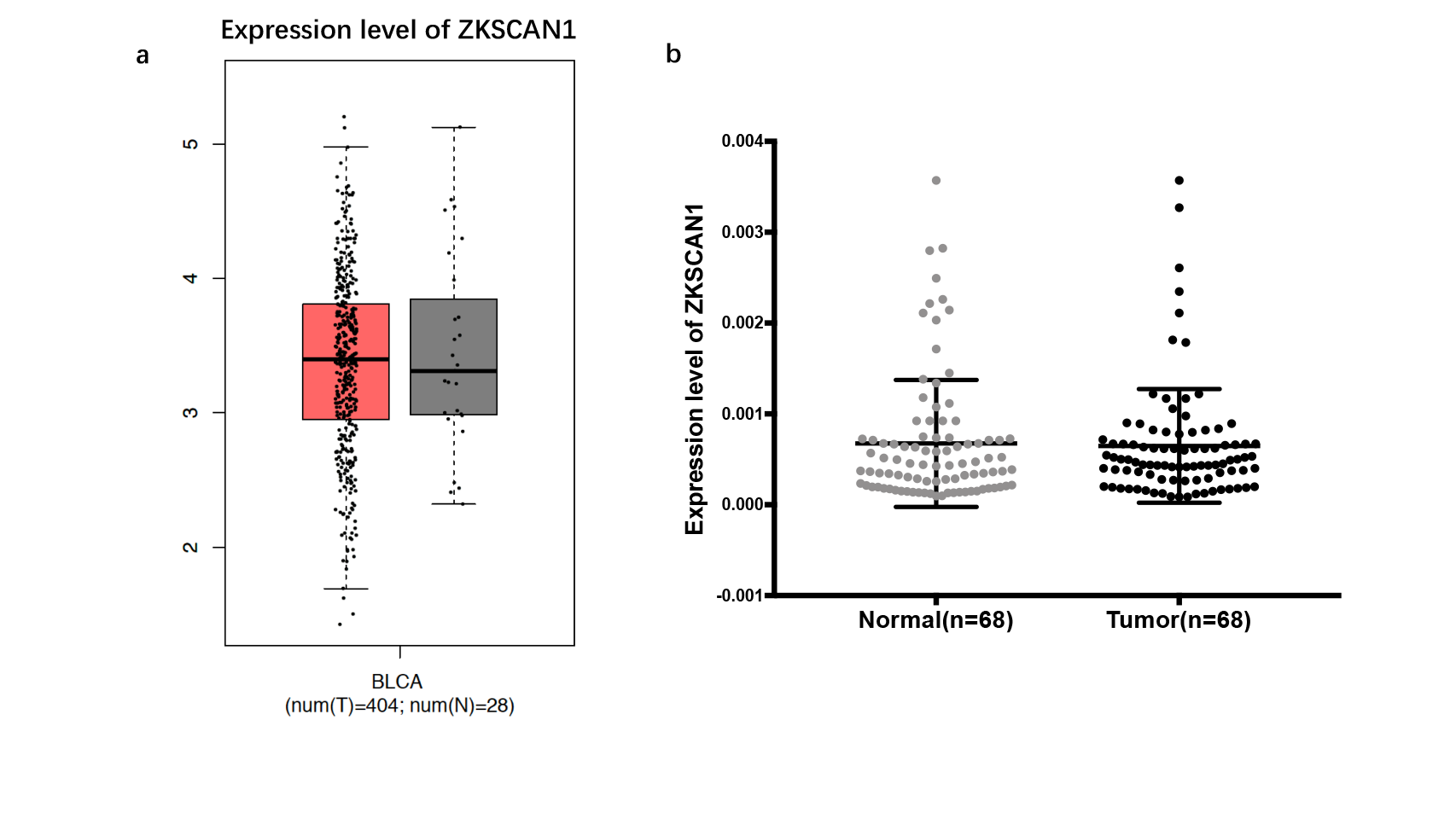

Supplement: Supplementary file 3 — Figure S1 Data from TCGA and our cohort showed the expression of ZKSCAN1 mRNA between BCa tissues and paired adjacent normal tissues. a Data from TCGA revealed that there was no significant changes in ZKSCAN1 mRNA between BCa tissues and matched adjacent normal tissues. b qPCR analysis for ZKSCAN1 mRNA in our cohort 1. (TIF 9877 kb) [file 12943_2019_1060_MOESM3_ESM.tif]

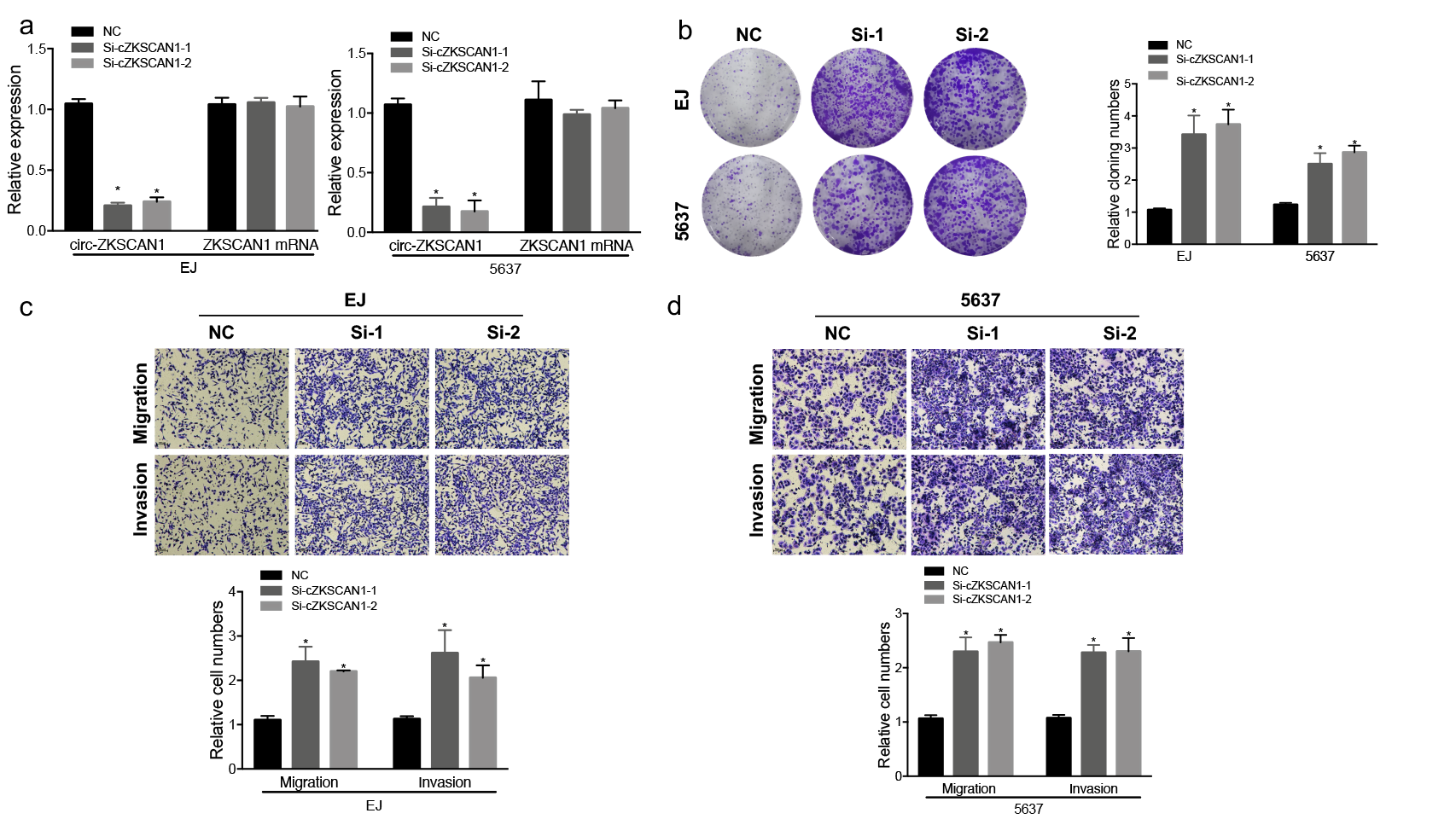

Supplement: Supplementary file 4 — Figure S2 circ-ZKSCAN1 silencing promotes proliferation, migration and invasion of BCa cells in vitro. a qPCR analysis for circ-ZKSCAN1 and ZKSCAN1 mRNA in EJ and 5637 cells treated with siRNAs or transfected with negative control. b-c The effect of si- circ-ZKSCAN1 on cell proliferation of EJ and 5637 cells was assessed by colony formation assays. d-e The cell migratory and invasive capabilities were examined in EJ and 5637 cells treated with circ-ZKSCAN1 siRNAs using transwell migration and invasion assays. (TIF 11349 kb) [file 12943_2019_1060_MOESM4_ESM.tif]

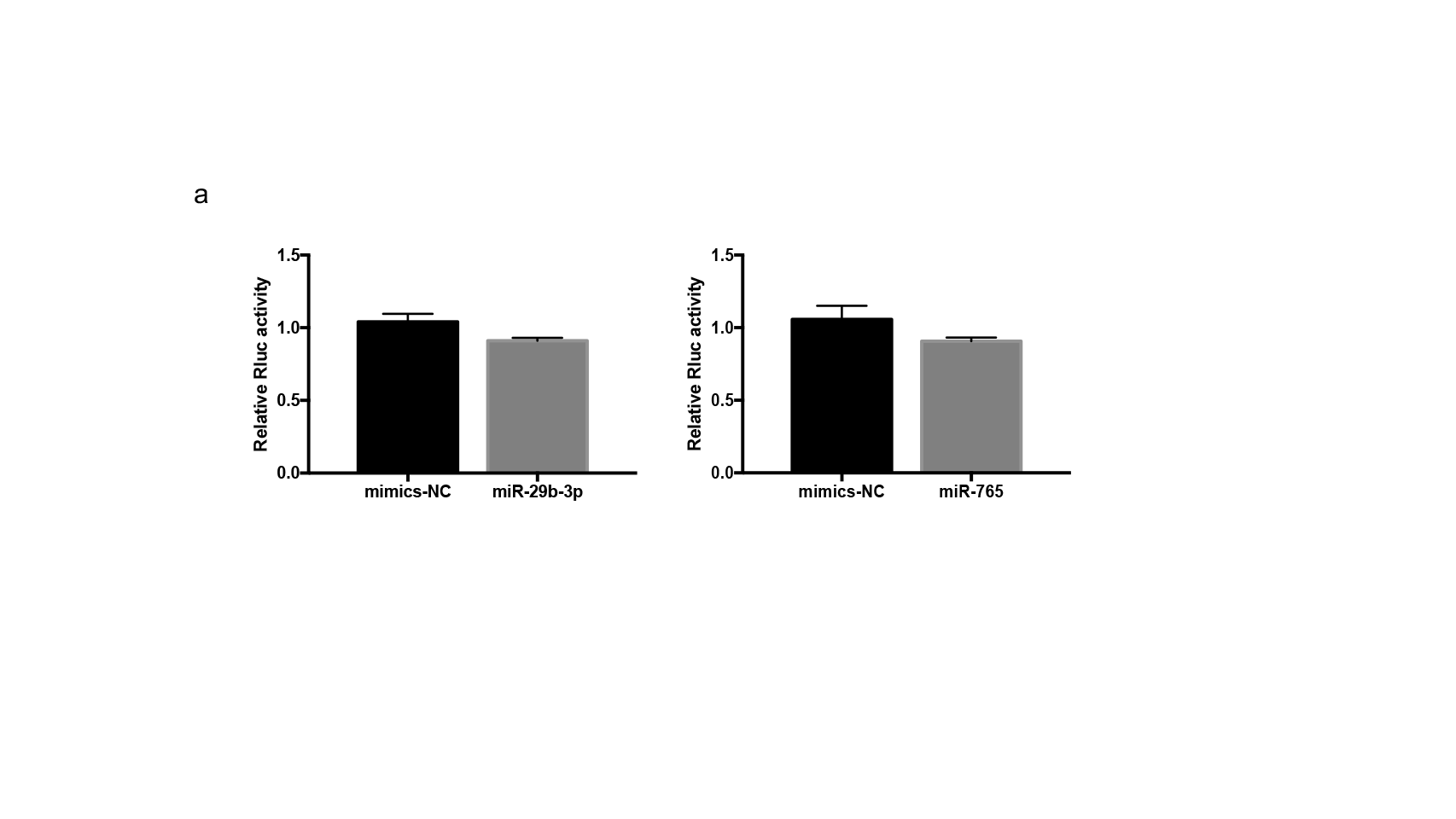

Supplement: Supplementary file 5 — Figure S3 Circ-ZKSCAN1 may function as a sponge of miR-1178-3p a The Renilla luciferase activity of wild type circ-ZKSCAN1 in the miR-29b-3p/miR-765 mimics or NC group. (TIF 3244 kb) [file 12943_2019_1060_MOESM5_ESM.tif]

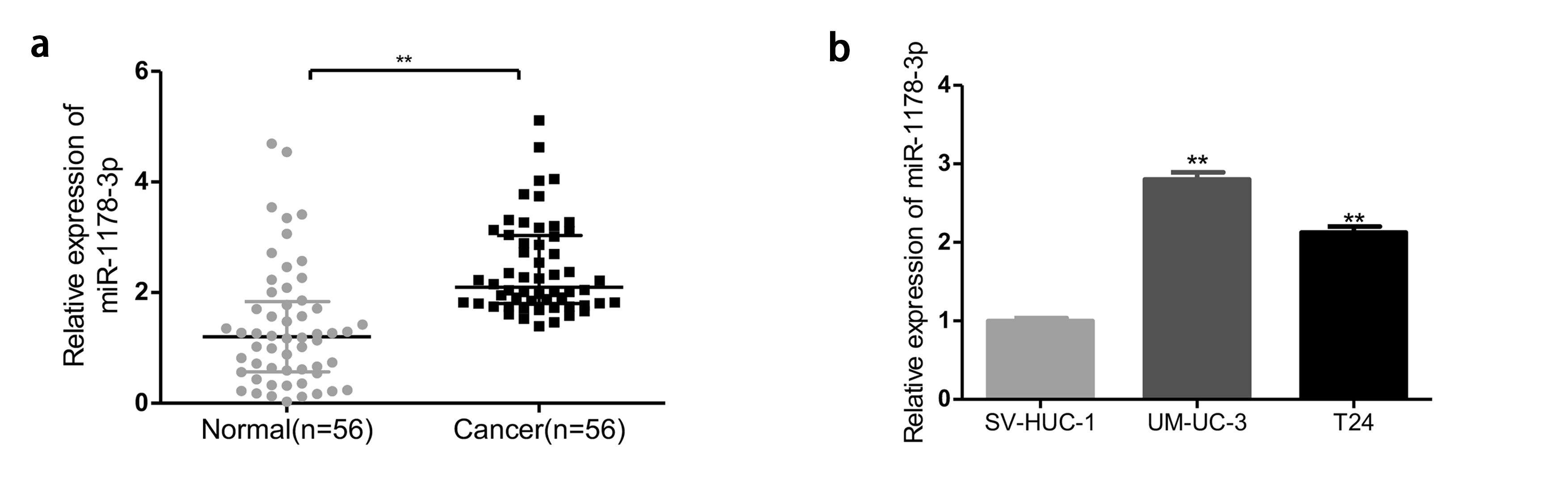

Supplement: Supplementary file 6 — Figure S4 MiR-1178-3p exerts an oncogenic role in BCa a-b qPCR revealed that miR-1178-3p was up-regulated in BCa tissues (n = 56) and cell lines (UM-UC-3 and T24), compared to the paired normal bladder tissues and SV-HUC-1. (JPG 447 kb) [file 12943_2019_1060_MOESM6_ESM.jpg]
